# Health Literacy and Complications in People With Type 2 Diabetes: An Exploratory Study

**DOI:** 10.7759/cureus.46064

**Published:** 2023-09-27

**Authors:** Mariana Cravo, Inês Rosendo, Luiz Miguel Santiago, Joana Abreu

**Affiliations:** 1 Family Medicine, Unidade de Saúde Familiar (USF) CampuSaúde, Golegã, PRT; 2 Family Medicine, Faculdade de Medicina de Coimbra, Universidade de Coimbra, Coimbra, PRT; 3 Family Medicine, Unidade de Saúde Familiar (USF) Conchas, Lisbon, PRT

**Keywords:** self-care, health knowledge, diabetes mellitus/complications, diabetes mellitus type 2, health literacy

## Abstract

Introduction: A person with diabetes is subject to developing micro and macrovascular complications and prevention requires an active role from the person. So, health literacy should have a preponderant role in the health of people with diabetes but this link is yet not fully understood. The objective of this study is to understand the relationship between health literacy and the prevalence of complications in people with diabetes mellitus type 2 (DM2).

Methods: This is a multicentric transversal observational exploratory study. A survey was conducted with two health literacy instruments, the Medical Term Recognition Test (METER) and Newest Vital Sign (NVS), filled out by people with DM2 coming to consultation in primary health centers in three main regions of Portugal.

Results: In this sample (n=141), 50.6% were male, 41 to 88 years old, and 56% earned more than the minimum wage. Using the METER tool, it was found that 57.4% of the diabetic patients had functional literacy. Adequate literacy was found in 24.1% with the NVS tool. Also with the NVS tool it was found that 36.2% of the sample subjects had s high probability of limited literacy. Utilizing the METER tool, a statistically significant decrease in health literacy was observed in individuals with diabetic complications (p=0.001). There was no significant relation between the presence of diabetic complications and present blood pressure values, low-density lipoprotein, and socioeconomic index.

Conclusion: In this study, we found a significant relation between lower health literacy and the presence of diagnosed DM2 complications (p=0.001).

## Introduction

Diabetes mellitus is considered one of the pandemics of the 21st century due to the constant increase in its incidence in recent years [1]. In Portugal, in 2015, the estimated prevalence of diabetes mellitus type 2 (DM2) was 13.3%, between the ages of 20 and 79 [2].

Diabetes, as a chronic disease, requires complex management of concepts and materials and, consequently, an important understanding of the disease by the people affected by it, so that they can take better care of themselves on a daily basis [3].

As a person with diabetes ages, there is an increased risk of developing complications: neuropathy, diabetic foot, retinopathy, nephropathy, and cardiovascular disease, these being the main causes of morbidity and mortality [2,4]. It is known that an increased risk of complications, besides being triggered by age and genetic susceptibility, can also be associated with poor control of intermediate indicators: high blood pressure, cholesterol, and poor glycaemic control, among others [4].

Thus, good metabolic and cardiovascular control are two of the main goals of a person with diabetes, as it is essential to prevent or delay the onset of these complications [1].

The emergence of complications is also related to relevant non-clinical factors, such as socioeconomic and psychosocial characteristics. Due consideration is also given to the important role of health literacy in the development of possible complications in people with diabetes, serving as a barrier to a better quality of life [4,5].

Health literacy has gained interest as a fundamental concept of the more active role of the person in their illness. It is known that there is a link between the level of literacy and the state of health of the person, probably because a low level of literacy is related to a more probable incomprehension of written and oral information provided by health professionals, inability to search services that may be necessary, or even following the indications of a prescription [6]. People with low literacy may not be able, for example, to interpret the information on a food label, or even read the information of a medicine [7]. In addition, literature links certain factors, such as age and academic education to a wide range of consequences on health care provision and results regarding health, which are associated with increased hospitalizations, and also a higher prevalence and severity of some chronic diseases [8-10].

Several studies confirm that low health literacy is associated with a decrease in knowledge about diabetes [8,11-14]. The development of micro and macrovascular complications such as retinopathy and cerebrovascular disease, have also been associated with reduced health literacy [7]. Another study shows that the risk of nephropathy increases as literacy decreases [15]. However, there are studies that do not find a significant association between health literacy and complications associated with diabetes [16].

This study aims to investigate the existence of a relationship between health literacy and complications of diabetes. Secondary objectives include understanding the relationship between complications of diabetes and sociodemographic variables and metabolic and cardiovascular control, in order to understand the influence of other associated factors.

## Materials and methods

After receiving a favorable opinion from the Ethics Committees of the Northern Regional Health Administration (No. 130/2017), Central Regional Health Administration (No. 59/2017), and Lisbon and Tagus Valley Regional Health Administration (No. 10299/CES/2017), a cross-sectional observational study began in July 2017.

The questionnaires were applied in a convenience sample in a primary care unit in 3 cities of Portugal (Unidade de Saúde Familiar Lagoa at Oporto; Unidade de Cuidados Saúde Personalizados Fernão de Magalhães at Coimbra; and Unidade de Saúde Familiar Conchas at Lisbon) to people with DM2 who went to a medical consultation in the days when data was collected, after being informed and signed consent. All patients with diabetes who came to the medical consultation on the data collection days were identified in each unit by their physicians and were then approached by the local main investigator who questioned their interest in responding to the questionnaire.

The anonymous questionnaire was composed of the Medical Term Recognition Test (METER) which evaluates if literacy is associated or not with word recognition and the Newest Vital Sign (NVS) aimed at a more functional assessment of literacy, both validated for Portugal and containing a request for informed consent [17,18]. In addition, it included the variables gender, age, academic training (number of years of schooling); the socioeconomic level evaluated by the socioeconomic drought index or SEDI (on a scale from 0 to 3, received less than the minimum wage - 1, schooling less than or equal to 4 years - 1, and lived alone - 1); disease progression time (years); glycated hemoglobin level (HbA1c); blood pressure; and most recent low-density lipoprotein (LDL).** **Complications diagnosed and recorded in the process (retinopathy, neuropathy, diabetic foot, nephropathy, and cardiovascular disease, specifically stroke and myocardial infarction) were also included. This data collection was done by inquiring users and checking the registrations in the clinical processes.

The sample size was calculated through the tool on the www.raosoft.com website, obtaining a minimum of 141 participants, with a confidence interval of 90% and a margin of error of 7%, defined for this exploratory study.

For the descriptive statistical and inferential analysis, not having normality on the distribution of the variables, the mean, minimal, and maximal values were calculated, and the Mann-Whitney U and Kruskal-Wallis non-parametric tests (p<0.05) were used to relate the complications registered with literacy in health and other variables.

## Results

Characterization of the sample

A total of 141 questionnaires were administered to people with DM2: 47 questionnaires in each unit. Table 1 represents the sociodemographic data of the total sample studied.

**Table 1:** Sociodemographic characteristics of the total sample (n=141) SEDI: Socioeconomic drought index; SD: Standard deviation

Variable		Values
Sample		n=141
Age (years)	Mean	68
Disease progression time (years)	Mean	8
Education (years)	Mean	4
SEDI	Mean±SD	1.20±0.97
Gender	Feminine/Masculine	70/71
Earns more or less than a minimal wage	More/Less	79/ 62
Lives alone	Yes/No	33/108

The sample, comprised of 141 respondents, encompassed people between 41 and 88 years of age (mean of 68 years), and 50.4% were men (Table 1). As for education, the mean was 4 years. Regarding the progression of the disease, the mean was 8 years, and for the most recent measurement of the HbA1c level was 6.6% (5.2-11.8%). The mean of the systolic blood pressure was 132 mmHg (89-167 mmHg), and the diastolic blood pressure was 78 mmHg (54-100 mmHg). Regarding LDL, the mean was 93 mg/dL (37.40-213.20 mmHg).

Through the METER instrument, it was possible to conclude that 57.4% (n=81) of the respondents have a functional literacy. When using NVS, adequate literacy was verified in 24.1% (n=34) of respondents (Table 2).

**Table 2:** Description of the sample according to the METER and NVS instruments METER: Medical Term Recognition Test; NVS: Newest Vital Sign

		No.	%
METER			
0-20	Low	10	7.1
21-34	Marginal	50	35.5
35-40	Functional	81	57.4
NVS			
0-1	High probability (50% or more) of limited literacy	51	36.2
2-3	Possibility of limited literacy	56	39.7
4-6	Adequate literacy	34	24.1

Regarding the prevalence of complications of diabetes in the respondents, 27.7% (n=39) presented diagnosed complications. Of this 27.7%, 6.4% (n=9) had retinopathy, 4.3% (n=6) had neuropathy; 1.4% (n=2) had diabetic foot and 12.1% (n=17) had nephropathy. Lastly, 6.4% (n=9) of respondents had already had an episode of stroke and 2.8% (n=4) of people had at least one acute myocardial infarction (AMI).

Association between literacy and diagnosed complications

Using the METER instrument, there was a significant correlation between the presence of complications and a lower average of literacy (p=0.001). Through the NVS instrument, the same relationship was also observed, but without statistical significance (p=0.283) (Table 3).

**Table 3:** Relationship between DM2 complications with the METER and NVS instruments METER: Medical Term Recognition Test; NVS: Newest Vital Sign; SD: Standard deviation; DM2: Diabetes mellitus type 2

	METER Mean±SD	NVS Mean±SD
With complications	2.21±0.70	1.77±0.78
Without complications	2.62±0.56	1.92±0.77
p-value	0.001	0.283

Correlation between complications and studied variables

In addition to health literacy, we looked for other variables related to the presence of complications and found only a significant relationship between the progression time of the disease and the presence of complications (p=0.001) (Table 4).

In terms of trends with no statistical significance, the presence of complications was associated with a more advanced age and a socioeconomic index tending to be higher (lower socioeconomic level) and education lower, when compared with individuals without complications. There has also been a tendency for patients who do not present complications to be those with lower average systolic blood pressure values, but higher diastolic blood pressure, HbA1c, and LDL.

**Table 4:** Correlation between complications and clinical-laboratory variables SEDI: Socioeconomic drought index; HbA1c: Glycated hemoglobin; LDL: Low-density lipoprotein; SD: Standard deviation

Variables	With complications Mean±SD	Without complications Mean±SD	p-value
Disease evolution time (years)	13.97±8.92	8.79±7.55	0.001
Age	68.92±9.36	66.48±10.53	0.258
SEDI	1.37±1.03	1.29±0.95	0.256
Education (number of schooling years)	5.44±3,25	7.25±4.34	0.019
Level of HbA1c	6.72±0.75	7.00±1.29	0.425
Systolic blood pressure (mmHg)	134.15±15.91	130.77±14.84	0.335
Diastolic blood pressure (mmHg)	75.69±10.26	77.34±8.95	0.406
LDL (mg/dL)	90.27±26.84	99.53±36.11	0.166

## Discussion

This exploratory study, to our knowledge, was the first in Portugal to evaluate the correlation between literacy and the development of complications of DM2.

A relationship between lower health literacy (METER) and the presence of complications (p=0.001) was found, but there was no significant association between the presence of complications and the socioeconomic level and education, nor with the values obtained in the variables of metabolic or cardiovascular control.

Several factors may explain the association between verbal recognition literacy and the complications of diabetes. People with low health literacy are associated with decreased knowledge about their disease [6-8,14]. Diabetes, as a chronic disease, demands some knowledge on the part of the individual. If it is scarce, the patient will increase the risk of developing complications [7,15,19]. Some studies also found a correlation between the presence of complications in diabetes and a more reduced literacy level, although other studies found no association [7,16,19].

A significant association was found between education and the presence of DM2 (p=0.019) complications, as confirmed by other literature about the correlation between high levels of education and high levels of health literacy [10].

It is known that the disease progression time is related to a higher incidence of complications in people with DM2 [2]. In this study, it has been found, as expected, that with an increase in the disease progression time, there is a higher incidence of complications (p=0.001).

Several studies have also related reduced literacy with sociodemographic factors, as triggers for the appearance of complications [19]. However, in this study, it was verified that the sociodemographic index was not significantly related to the presence of complications (p=0.256), which may indicate that health literacy may be a decisive factor in complications of diabetes, regardless of the sociodemographic level of those people.

Comparing this sample with the sociodemographic data regarding the Portuguese population with DM2, a similar degree of similarity was obtained since the mean age is 68 years [2]. Among the most prevalent complications in the Portuguese population are cardiovascular disease (stroke, 6.4%, and AMI, 2.8%), and nephropathy (12.1%) [2]. These are also the ones with the highest incidence in this study. However, diabetic foot (1.4%), retinopathy (6.4%), and neuropathy (4.3%) were underrepresented.

Regarding the clinical and laboratory parameters, it was observed that the average diastolic blood pressure was under better control than the systolic blood pressure, which has been observed in other studies [20]. In this sample, it was found that HbA1c levels were lower in people with complications, although not a significant difference. This may be related to greater care and tight control in this population, either because of a greater motivation to change their lifestyle and adherence to therapy after having undergone some complications, or through more intensive and rigorous treatments and consultations after the development of complications. The fact that these variables do not have an association with the presence of complications will lead one to think that the influence of health literacy on the emergence of complications may be independent of the control of these clinical parameters, besides the influence of the socioeconomic level.

As for limitations, we utilized data derived from three distinct locales spanning diverse geographical regions. Although diversified, the sample was of convenience and limited in the number of respondents because it was an exploratory study, which limited the generalization to the Portuguese population and the analysis of some variables. Given the urban nature of the cities where the study was conducted, which inherently presume a higher literacy rate, the intrinsic potential for selection bias is evident, as this sampling does not adequately mirror the rural population.

It is prudent to note further that the use of questionnaires to gauge behaviors raises the possibility of inaccuracies and biases. Furthermore, the willingness and motivation to respond to a questionnaire may in themselves be deemed indicators of health literacy, consequently potentially resulting in the exclusion of less proficient segments within the scope of our study.

Another limitation of the study was due to the fact that the individual medical records of the respondents were not updated, nor were the complications properly recorded and coded.

Given that there were two researchers in the data collection, this may have led to an inter-observer bias. Moreover, since it is a cross-sectional study, it is not possible to determine causality among these variables.

Despite this, it must be underscored as a merit of this current work the adoption of a population-based sample, which facilitated the inclusion of individuals with diabetes across various stages of the condition. Also, the present study is made up of other points that reinforce its robustness: it encompasses three Portuguese cities in different regions, which diversifies the sample and, although it is of convenience, no selection was made when choosing the respondents that met the inclusion criteria of the study, except for the fact that they had a medical consultation on the days the data was collected; this study considers two assessment tools for health literacy validated in Portugal, which allow a more global assessment of the capacities of the respondents, also encompassing the most frequent complications in the Portuguese population, taking into account intermediate risk factors (blood pressure, glycaemic control, among others) and socioeconomic factors, in order to understand its possible influence in health literacy and complications of diabetes. Risk and socioeconomic factors have significant implications for health literacy, which pertains to an individual's capacity to access, comprehend, and effectively employ health-related information to make informed decisions about their well-being. The interplay between these factors impacts health literacy in several ways. For example, individuals with higher socioeconomic status tend to be more exposed to health-promoting environments, preventive care, and health risk awareness. Conversely, those with lower socioeconomic status may lack knowledge about health risks and the significance of health-promoting behaviors. Also, reduced education can impair reading, numeracy, and critical thinking skills necessary for comprehending intricate health information.

Lastly, the study was not based on the complications reported by the people, but on the data collected in the respective clinical records, being more objective when compared with studies that were based on what the respondent reported [7].

It is important to point out that this project is an exploratory study of a poorly explored subject, namely in a population with low health literacy, such as the Portuguese population. It seems important to continue its development, with larger samples, in order to be more representative of this population. It would also be important to try and understand how literacy is influencing the relation with complications and what factors are being conditioned by this low health literacy (medication adherence, non-pharmacological therapy, empowerment), to help determine the best way to intervene on this issue, as to improve the quality of life and reduce mortality in this population.

## Conclusions

Since the objective of this study was to understand if there is a relationship between health literacy and the presence of complications of diabetes mellitus type 2 (DM2), it was possible to conclude that in this sample there is a significant relationship between a more limited literacy and a higher incidence of complications associated with DM2 (p=0.001).

It was also concluded that there is no significant relationship between the presence of complications with systolic and diastolic blood pressure, low-density lipoprotein, and the socioeconomic index, so that the relationship between literacy and complications may be independent of these variables. 

Future research should explore the development and effectiveness of health literacy educational programs, potentially mitigating the incidence of complications. It would be important to invest in longitudinal studies to explore the temporal relationship between changes in health literacy levels and the development of complications over time. Additionally, there is potential for conducting interventional studies to further validate the role of health literacy in complication prevention.
